# Semaphorin 3C and Its Receptors in Cancer and Cancer Stem-Like Cells

**DOI:** 10.3390/biomedicines6020042

**Published:** 2018-04-08

**Authors:** Jing Hao, Jennifer S. Yu

**Affiliations:** 1 Department of Stem Cell Biology and Regenerative Medicine, Cleveland Clinic, Cleveland, OH 44195, USA; haoj@ccf.org; 2 Department of Radiation Oncology, Cleveland Clinic, Cleveland, OH 44195, USA; 3 Cleveland Clinic Lerner College of Medicine, Cleveland Clinic, Cleveland, OH 44195, USA

**Keywords:** semaphorin, plexin, neuropilin, Sema3C, Semaphorin 3C, cancer stem cell, glioma stem cell, invasion, migration, angiogenesis, neurodevelopment, axon guidance, glioma, glioblastoma, cancer

## Abstract

Neurodevelopmental programs are frequently dysregulated in cancer. Semaphorins are a large family of guidance cues that direct neuronal network formation and are also implicated in cancer. Semaphorins have two kinds of receptors, neuropilins and plexins. Besides their role in development, semaphorin signaling may promote or suppress tumors depending on their context. Sema3C is a secreted semaphorin that plays an important role in the maintenance of cancer stem-like cells, promotes migration and invasion, and may facilitate angiogenesis. Therapeutic strategies that inhibit Sema3C signaling may improve cancer control. This review will summarize the current research on the Sema3C pathway and its potential as a therapeutic target.

## 1. Introduction

The connection between neural networks and cancer has long been recognized. In almost all solid tumors, perineural invasion is recognized as an important adverse prognostic feature. This suggests that cancers have evolved mechanisms to grow and spread along nerves or recruit nerves along which they can proliferate and migrate. More recent data reveal that neural stimulation can trigger a release of neurotransmitters that contribute to the growth, differentiation, and proliferation of tumor cells and cancer stem-like cells (CSCs) [1]. Indeed, many neural developmental programs are hijacked by cancer cells to promote their own growth, survival, and invasion. These programs include axonal guidance proteins and their receptors, notably the Eph/ephrin [2], Slit/Robo [3], neurotrophin [4], Netrin/DCC/UNC5 [5,6], and Semaphorin/Neuropilin/Plexin families of proteins [7,8,9,10,11,12,13].

Intriguingly, these axonal guidance systems not only promote cancer growth themselves, but also promote migration and angiogenesis. Considering that in development, the nervous system and blood vessels grow together in parallel, it is not surprising that these neurodevelopmental programs also help to shape the vasculature and that the nervous and cardiovascular systems share signaling pathways. In this review, we will discuss the role of Semaphorin 3C (Sema3C) as an example of one axonal guidance cue that drives cancer progression.

The Semaphorin protein family is a large family of axon guidance molecules that was first found to help shape the developing nervous system [14]. The proper wiring of the nervous system requires that axons be guided to their targets with high precision to form proper synapses. Semaphorins serve as cues for axons to navigate through their environment, with some semaphorins serving as attractants and others serving as repellants. Proteins in the semaphorin family all share a common sema domain and a plexin-semaphorin-integrin (PSI) domain. There are more than 20 semaphorins divided into 8 classes that are found in invertebrates, vertebrates, and viruses [13,15]. These semaphorins include transmembrane proteins, membrane-anchored proteins, and secreted proteins. In general, semaphorins dimerize and bind to a pair of neuropilins which recruit a pair of plexin co-receptors. There are two neuropilins, Nrp1 and Nrp2, and nine plexin co-receptors that are divided into four classes. Sema3C binds to Nrp1 and Nrp2 with similar affinity [7]. Semaphorin 3E (Sema3E) is the only semaphorin that has been shown to bind directly to a plexin receptor and activate signaling independent of the neuropilins [16]. Considering the numerous combinations of semaphorins, neuropilins, and plexins, a large number of cellular responses can be generated. Sema3C and its receptors are frequently overexpressed in cancer and are associated with invasion and metastasis. In this review, we will introduce the role of Sema3C signaling in development and focus on the role of Sema3C and its receptors in oncogenesis and in CSCs.

## 2. Sema3C Function in Development

Class 3 semaphorins are secreted proteins whose spatial distribution across gradients can counterbalance each other to fine tune cellular responses [17]. In neurodevelopment, opposing gradients of Sema3C and Sema3A expression help to guide axons to their target [18]. Sema3C functions as an axon attractant in migrating cortical axons, whereas Sema3A functions as a repellant [18]. Sema3C also promotes axonal growth of dopaminergic neurons in humans and rats [19,20], is essential for cortical and hippocampal neuron polarization and migration [21,22,23], and helps guide motor neurons to their targets [24,25], revealing a diverse range of neuronal projections that Sema3C regulates.

Sema3C is also implicated in the development of the enteric nervous system. Loss of function mutations of Sema3C are found in patients with Hirschsprung disease, a congenital disease in which the enteric nervous system fails to form in parts of the intestine [26]. In Crohn’s disease, which is an inflammatory bowel disease, increased Sema3C expression in intestinal crypts correlates with a reduction in mucosal sympathetic nerve fibers [27]. The loss of sympathetic nerves was thought to promote inflammation.

Interestingly, Sema3C knockout mice do not appear to have defects in their nervous system [28]. Instead, these mice exhibit cardiovascular deficits, including cardiac outflow tract and aortic arch defects. Knockout mice are cyanotic and die within 24 h after birth. The failure of cardiac neural crest cells to migrate properly results in improper septation of the cardiac outflow tract [28]. The penetrance of the phenotype is largely dependent on the strain of mouse, with the CD1 background strain exhibiting the greatest penetrance [28].

The mechanisms behind these developmental abnormalities have been examined using tissue-specific and ligand-specific mouse mutants. Neural crest cells secrete Sema3C which binds to Nrp1 in endothelial cells to promote endothelial-to-mesenchymal transition, and these dedifferentiated cells and neural crest cells participate in septation of the outflow tract [29,30]. Others have shown that expression of attractive cues from Sema3C and PlexinD1 and repulsive cues from Sema3D, Sema6A, Sema6B, and PlexinA1 guide the migration of cardiac neural crest cells [31,32]. Further supporting a role for Sema3C in cardiac development comes from genetic analysis of patients with persistent truncus arteriosus (PTA). These patients have mutations in the transcription factor GATA6, which is unable to transactivate Sema3C and its receptor PlexinA2 [31,33]. Other transcription factors, including Foxc1, Foxc2, and Tbx1, participate in cardiac outflow tract development by regulating Sema3C expression [34,35,36]. Mutation of PlexinD1 is also found in truncus arteriosus [36].

**Figure 1:**
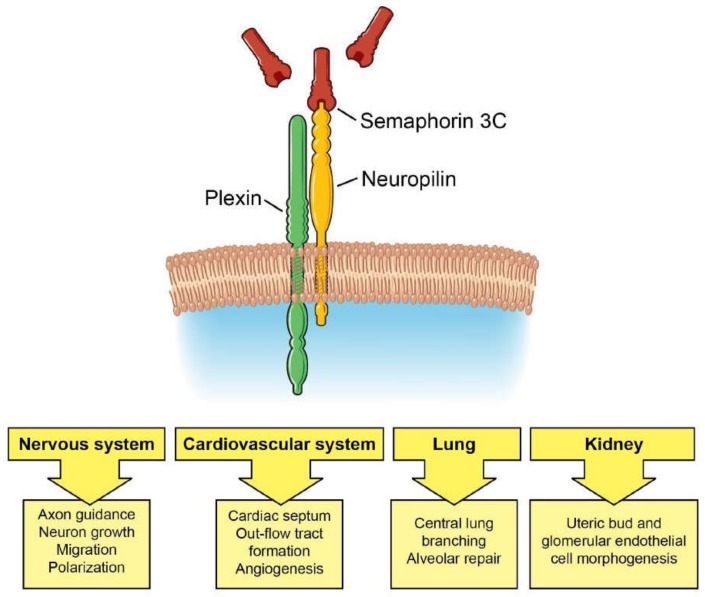
Sema3C/Neuropilin/Plexin signaling in development. Sema3C signaling guides development of the nervous and cardiovascular systems, lung, and kidney.

Sema3C and Sema3A also play important roles in the development of many other organs. In lung development, Sema3C stimulates lung branching morphogenesis in the central lung, whereas Sema3A reduces branching in the distal mesenchyme in the lung periphery, thereby guiding proper branching [37]. Interestingly, in a rat bronchopulmonary dysplasia model, Sema3C treatment reduced inflammation and apoptosis to maintain alveolar and lung vascular growth, suggesting that Sema3C may facilitate lung repair [38]. In kidney development, Sema3C and Sema3A similarly help to shape renal ureteric bud development and formation of the glomerular filtration unit [39,40]. Sema3C promotes ureteric bud and glomerular endothelial cell morphogenesis, whereas Sema3A negatively regulates these processes. Thus, the spatial distribution and gradients of expression of Sema3C and Sema3A across an organ regulate proper tissue patterning. The roles of Sema3C in development are summarized in Figure 1.

## 3. Sema3C in Cancer and Cancer Stem-Like Cells

Sema3C plays an oncogenic role in many different types of cancers. Sema3C is an indicator of poor prognosis and progression of glioblastoma, prostate cancer, breast cancer, liver cancer, gastric cancer, pancreatic cancer, and lung cancer [11,41,42,43,44,45,46,47,48]. Whether other semaphorins cooperate with or compete with Sema3C in cancer is not well understood.

In gliomas, Sema3C and its receptors are overexpressed in human glioma cell lines [49]. Sema3C is overexpressed in 85% of glioblastoma and its receptors PlexinA2 and PlexinD1 are detected in all glioblastoma specimens analyzed [50]. Analysis of human brain tumor samples revealed that Sema3C protein levels are markedly increased in glioblastoma (grade IV) compared to grade I–III astrocytomas, and this increase in Sema3C expression was associated with shorter survival time [51]. Additionally, Sema3C has been found to be one of the top 20 most frequently altered genes in glioblastoma [52]. These genetic changes include amplification and missense mutations [52].

Cancer stem-like cells (CSCs) promote cancer progression due to their capacity for self-renewal and invasion and intrinsic ability to repair DNA damage [53,54,55]. In glioblastoma, Sema3C functions as a survival and invasion cue for glioma stem-like cells (GSCs) [50]. Sema3C and its receptors PlexinA2 and PlexinD1 are coordinately expressed by GSCs, and together, they form a feed-forward autocrine and paracrine loop to stimulate GSC survival and invasion [50]. Sema3C binding to the Nrp1/PlexinA2/PlexinD1 receptor complex was found to activate Rac1/NF-κB signaling to promote the survival and migration of GSCs [50]. In GSCs, both PlexinA2 and PlexinD1 co-receptors are needed to transduce Sema3C signals as knockdown of either one of the plexins induces apoptosis. In GSCs, signaling through two different types of plexins in the receptor complex, as opposed to the more usual plexin homodimer, adds an additional layer of complexity to semaphorin signaling.

In glioblastoma, Sema3C was selectively expressed in GSCs but not in their counterpart neural progenitor cells or non-stem tumor cells [50]. Forced differentiation of GSCs resulted in loss of Sema3C expression. This suggests that GSCs have independently evolved a way to reactivate Sema3C expression, and upon differentiation, Sema3C expression is turned off. These studies demonstrate a key role for Sema3C in maintaining GSCs and identify Sema3C as an important therapeutic target in glioblastoma [50].

In prostate cancer, increased expression of Sema3C strongly correlates with biochemical recurrence [56] and castration resistance [48]. Overexpression of Sema3C in prostate cancer cell lines enhances invasion [57] and facilitates stem cell marker expression and tumorsphere formation, suggesting a role for Sema3C in maintaining prostate CSCs [47]. Further stem cell assays are needed to confirm the function of Sema3C in prostate CSCs. Sema3C overexpression contributes to resistance to androgen deprivation therapy through activation of multiple growth factor receptors, including epidermal growth factor receptor (EGFR), ErbB2, and Met through PlexinB1 [48]. Expression of Sema3C in prostate cancer is induced by the androgen receptor and GATA2 and negatively regulated by FOXA1 [46]. Increased expression of Sema3C in prostate cancer tissues can also be attributed to hypomethylation of its promoter [58]. These studies support a role for Sema3C in prostate cancer progression and potentially in prostate CSC maintenance.

In breast cancer, Sema3C appears to promote tumor progression. Sema3C is expressed more highly in triple-negative and Her2-positive breast cancer, two aggressive subtypes that are highly metastatic [45]. In breast cancer cell lines, Sema3C depletion reduces cell proliferation and migration [59,60]. Cleavage of Sema3C by the metalloproteinase ADAMTS1 facilitates its release from the extracellular matrix to bind its receptors on cancer cells to promote cell migration [61]. Another report suggests that furin cleavage activates Sema3C [62]. In support of this, a furin-resistant form of Sema3C impairs lymphangiogenesis and reduces metastasis [62]. Because furin is expressed in many tumors, Sema3C is likely processed into its active form to promote tumor progression.

Neuroblastoma is a pediatric cancer derived from sympatho-adrenal neural crest cells. In contrast to epithelial cancers, Sema3C serves as a cohesion cue in neuroblastoma [63]. Downregulation of either Sema3C or its receptor PlexinA4 induces neuroblastoma dissemination [63]. Signaling was dependent on Nrp1 and Nrp2, as inhibition of both Nrp receptors was required to promote metastasis. Together, these studies indicate that Sema3C functions in a cell-type and context-dependent manner.

Crosstalk between tumor cells and blood vessels mediated by Sema3C may also modulate tumor progression. The role of Sema3C in modulating angiogenesis is incompletely understood. Blood vessels are needed to support growing tumors, and Sema3C, Nrp1, and PlexinD1 play important roles in shaping the vasculature in development [64]. Knockout mice of Sema3C, Nrp1, and PlexinD1 exhibit similar cardiovascular defects [28,64,65]. In tumors, the role of Sema3C is less clear, with some reports supporting that Sema3C promotes angiogenesis and other reports suggesting that it inhibits angiogenesis. In glioblastoma, Sema3C-positive cells were found in the perivascular niche where GSCs are known to reside [50]. Whether Sema3C secreted by GSCs recruited endothelial cells, which express Sema3C receptors, to their vicinity is not clear. In breast cancer, Sema3C expression correlates with increased microvessel density, but in oral cancer, Sema3C levels inversely correlates with microvessel density [45]. As discussed above, expression of a furin-resistant mutant of Sema3C inhibits lymphangiogenesis and reduces metastatic spread of a triple-negative breast cancer cell line [62]. Exogenous administration of either wild-type or furin-resistant Sema3C can reduce pathologic neoangiogenesis in the retina by inducing apoptosis of endothelial cells in immature microvessels [66,67].

The mechanism by which Sema3C regulates angiogenesis is complex. Sema3C binding to Nrp1 or Nrp2 and PlexinD1 on endothelial cells can promote angiogenesis [64]. Nrp1 can also interact with vascular endothelial growth factor receptor (VEGFR) and modulate its responsiveness to vascular endothelial growth factor (VEGF) [68,69]. Sema3C binding to Nrp1 or Nrp2 may reduce the binding of VEGF to the VEGFR/Nrp1 receptor complex, thereby serving as a competitive inhibitor of VEGF [66]. Class 3 semaphorins have also been found to modulate the immune microenvironment (reviewed in [70]), but the contribution of Sema3C in mediating this process is unclear. More studies are needed to dissect the role of Sema3C in modulating the tumor microenvironment.

**Figure 2:**
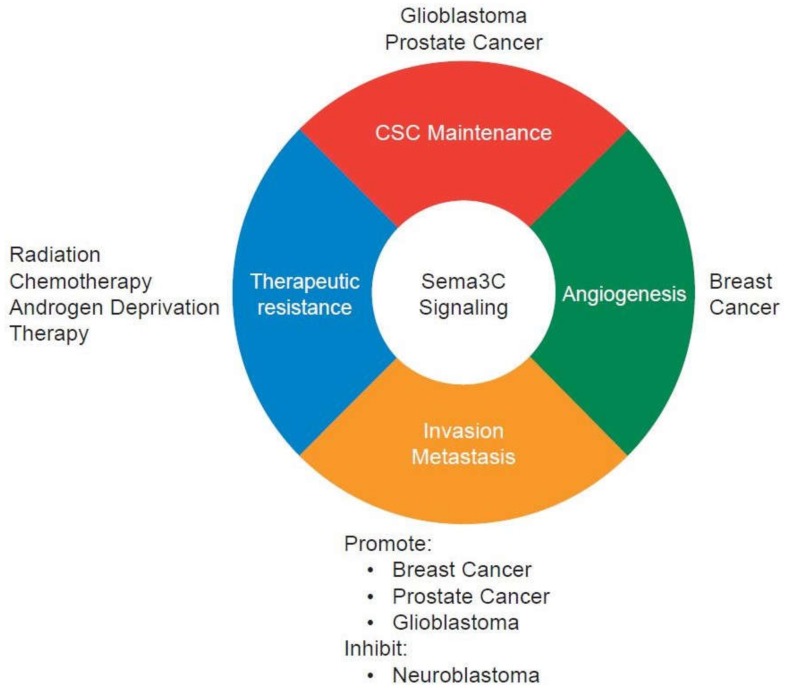
Context-dependent functions of Sema3C signaling in cancer progression. Sema3C signaling is implicated in cancer stem-like cell (CSC) maintenance, angiogenesis, invasion, metastasis, and therapeutic resistance. In breast cancer, furin-cleaved Sema3C is the active form and promotes tumor invasion, metastasis, and lymphangiogenesis.

Sema3C is identified as a drug resistance gene in cancer cell lines [71]. Sema3C is overexpressed in cisplatin-resistant ovarian cancer cell lines. Sema3C confers resistance not just to chemotherapy but also X-ray and UV irradiation. These data suggest that inhibition of Sema3C signaling may sensitize cancer cells to cytotoxic therapy. It would be interesting to explore the role of Sema3C in therapeutic resistance in other cancer types. The roles of Sema3C signaling in cancer progression are summarize in Figure 2.

## 4. Sema3C Receptors in Carcinogenesis

Both neuropilins and plexins have been implicated in cancer (reviewed in [7,8,72,73,74]). As Sema3C signaling has been implicated in GSC maintenance, we will focus our discussion on its receptors in glioblastoma. Elevated Nrp1 expression is a risk factor for glioblastoma recurrence and shorter patient survival [75]. Disruption of Nrp1 signaling by siRNA knockdown significantly inhibits proliferation of glioma cell lines [76] phenocopying Sema3C knockdown.

Nrp1 has multiple binding partners, including EGFR, platelet-derived growth factor receptor (PDGFR), VEGFR, p75 neurotrophin receptor, integrins, TGFβRII, and L1CAM [68,77,78,79,80,81,82]. Binding of Sema3C or other semaphorins to Nrp1 may modulate these signaling pathways. For example, Nrp1 serves as a receptor of VEGF and modulates VEGF binding to VEGFR2 [83]. VEGFR2 is preferentially expressed in GSCs. An autocrine VEGF/VEGFR2/Nrp1 signaling loop contributes to GSC maintenance [78]. Since GSCs express high levels of Sema3C, and Sema3C binds to Nrp1, Sema3C may modulate VEGF interaction with the VEGFR2/Nrp1 receptor complex. Temozolomide, the standard chemotherapy for glioblastoma, induces apoptosis of glioblastoma cell lines in part through downregulation of Nrp1, and temozolomide synergizes with anti-angiogenic treatment to further induce cell death [78,84,85].

The role of Nrp2 in glioblastoma is controversial. One study supports that Nrp2 expression is low in both low- and high-grade gliomas [86] while another study suggests that Nrp2 is expressed highly in 37% of glioblastoma and that its concomitant overexpression with VEGF-C correlates with poor patient survival [87]. The extent to which Sema3C or other ligands that bind Nrp1 and Nrp2 modulate response to chemotherapy and anti-angiogenic therapy warrants further investigation.

The role of plexins in glioblastoma has not been fully investigated. In GSCs, Sema3C requires both PlexinA2 and PlexinD1 to transduce Sema3C pro-survival signaling [50]. PlexinD1 is also expressed in tumor-associated blood vessels [88], and it is possible that secretion of Sema3C from GSCs in the perivascular niche can recruit and communicate with endothelial cells. Indeed, secretion of soluble factors, such as VEGF, from GSCs promotes angiogenesis [89], and GSC-endothelial cell crosstalk reinforces the stem cell phenotype and resistance to radiotherapy [90].

## 5. Therapeutic Strategies Targeting Sema3C and Its Receptors

In many tumors, Sema3C appears to promote cancers and in particular CSC survival. In glioblastoma, Sema3C does not appear to be expressed in neural progenitor cells, yet is highly expressed in GSCs [50]. Conversely, PlexinA2 and PlexinD1 are used by neural progenitor cells, likely as receptors for other semaphorins, and knockdown of PlexinA2 or PlexinD1 in these cells induces apoptosis. Together, these data suggest that targeting Sema3C in glioblastoma may have a favorable therapeutic ratio. In GSCs, Rac1 appears to be a critical signaling hub activated by Sema3C to promote survival and invasion [50]. GSCs are more sensitive to Rac1 inhibition compared to neural progenitor cells [50], suggesting that targeting Rac1 may also have a large therapeutic window. Additionally, as Sema3C signaling promotes GSC survival through NF-κB pro-survival signaling, antagonizing Sema3C or Rac1 may also sensitize GSCs to radiation and chemotherapy.

In prostate cancer, Sema3C appears to modulate multiple mitogenic pathways [48]. Combined targeting of Sema3C and these other pathways may reduce resistance mechanisms. Given the role of Sema3C in androgen resistance, targeting Sema3C offers promise for patients with castration-resistant prostate cancer, who have few therapeutic options. As seen in neuroblastoma, Sema3C may be tumor-suppressive and suppress metastasis. Therefore, strategies that inhibit Sema3C signaling will need to be examined in the proper context.

Targeting neuropilins may also have therapeutic value and has been the subject of excellent reviews [72,91]. Treatment of animal models of glioblastoma and breast cancer with peptides encoding the neuropilin transmembrane domain that interfere with receptor dimerization resulted in reduced tumor burden, metastasis, and angiogenesis [92,93]. Further work on antagonizing Sema3C or its receptors in the context of anti-angiogenic therapy and other targeted therapies is needed.

The quest to develop small molecule inhibitors targeting Sema3C or its receptors has not yet been fruitful [73]. However, specific inhibitors of Sema3A, xanthofulvin and vinaxanthone, have been isolated from the broth of fermented fungus [94,95,96]. Synthesis of these compounds and their derivatives has shown promise in facilitating axonal regeneration after injury in model organisms [97,98]. Similar approaches may be used to find inhibitors of Sema3C signaling. Strategies that take into consideration cell type and cellular context are needed to translate anti-Sema3C therapies into the clinic. 

## 6. Future Perspectives

The current literature supports an oncogenic role of Sema3C and its receptors in most malignancies. Despite the data collected from patients and animal models, there are still some important questions to be answered. What is the extent of crosstalk between Sema3C signaling and other oncogenic pathways in cancer cells themselves, blood vessels, immune cells, and other stromal cells? How does Sema3C maintain the stemness of GSCs and other CSCs? How does Sema3C signaling contribute to therapeutic resistance? What is the role of Sema3C in guiding tumor angiogenesis and creation of the perivascular niche? A more comprehensive understanding of Sema3C signaling in cancer will guide us in integrating anti-Sema3C therapies into clinical care.
